# Supplementary material for: A broadband achromatic polarization-insensitive metalens consisting of anisotropic nanostructures
Source: Nat Commun. 2019 Jan 21;10:355. doi: 10.1038/s41467-019-08305-y (PMC6341080; doi:10.1038/s41467-019-08305-y)
Supplement: Supplementary file 2 — Supplementary Information [file 41467_2019_8305_MOESM2_ESM.pdf]

Supplementary Information for:

A broadband achromatic polarization-insensitive metalens consisting of anisotropic nanostructures

Wei Ting Chen[1,*], Alexander Y. Zhu[1], Jared Sisler[1,2], Zameer Bharwani[1,2] and Federico Capasso[1,*]

[1]*Harvard John A. Paulson School of Engineering and Applied Sciences, Harvard University, Cambridge, Massachusetts 02138, USA*

[3]*University of Waterloo, Waterloo, ON N2L 3G1, Canada*

*Corresponding author: capasso@seas.harvard.edu and weitingchen@seas.harvard.edu

Simulated focusing efficiency for an achromatic metalens

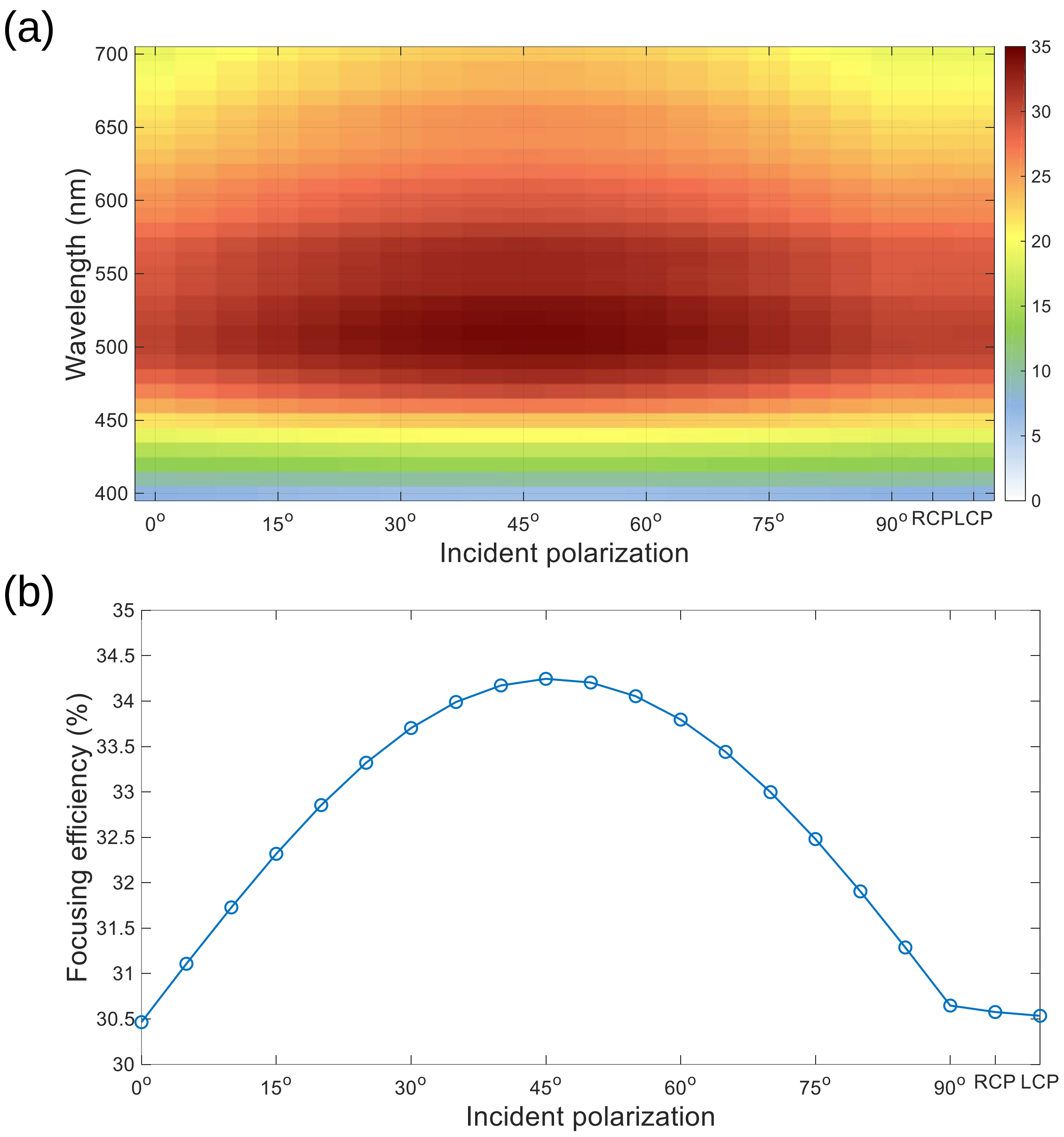

Supplementary Figure 1: FDTD simulation results for the achromatic and polarization-insensitive metalens' focusing efficiency shown in Supplementary Movie. (a) Focusing efficiency (colors) as a function of wavelength and incident polarization. (b) Focusing efficiency at incident wavelength $\lambda = 500$ nm. It shows a slight variation in focusing efficiency under different incident polarizations, which is in line with our experimental data shown in Fig. 3(d) in the main text. This simulation was performed by simulating a metalens with a 7.5 μm diameter using an FDTD solver (Lumerical, Inc). The metalens has a numerical aperture of 0.6.

Focal intensity profiles

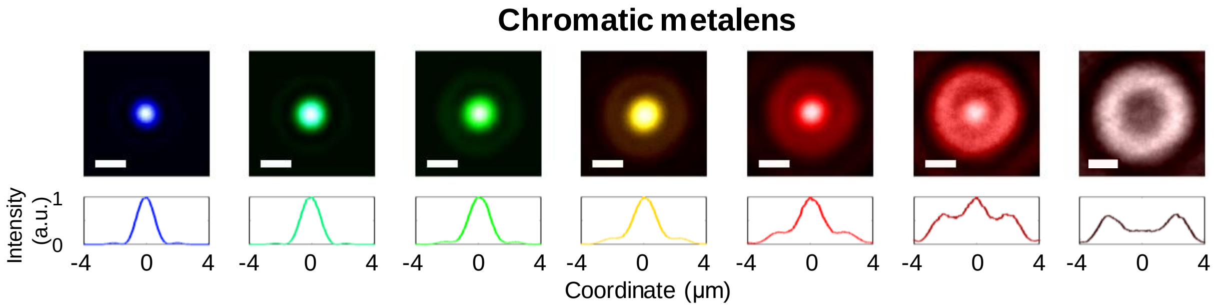

Supplementary Figure 2: Normalized intensity profiles for the chromatic metalens along the white dashed lines of Fig. 3(a).

Focal spot analysis

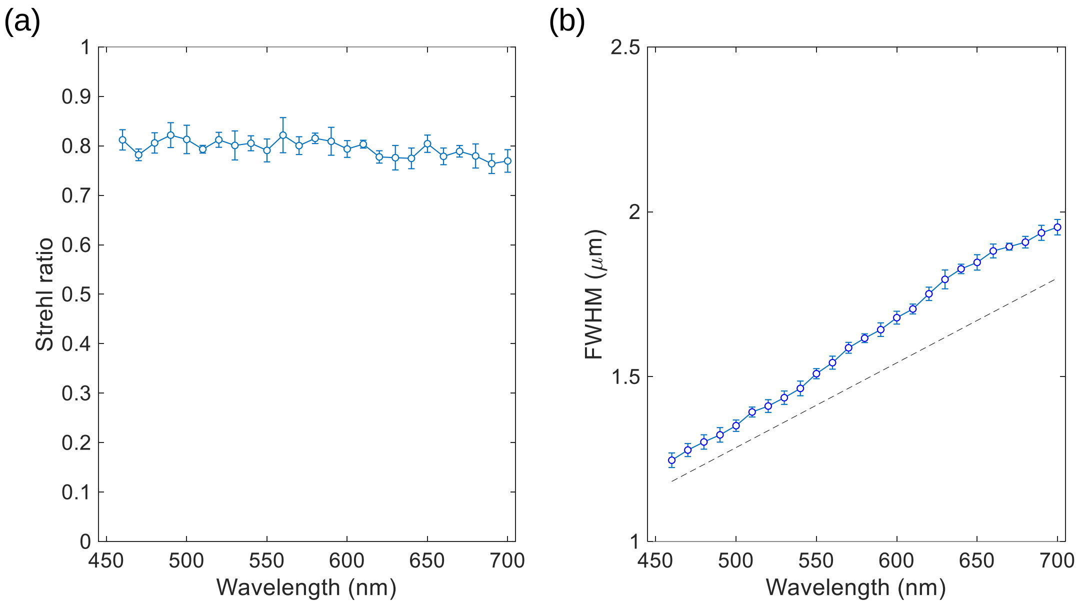

Supplementary Figure 3: Strehl ratios and focal spot sizes of an achromatic and polarization-insensitive metalens. (a) Strehl ratio as a function of incident wavelength. The Strehl ratio was calculated by dividing the peak intensity of the measured focal spot with that of a theoretical Airy disk, assuming their power summations to the second lobe are equal. (b) Full-width at half-maxima of measured focal spots. The black dashed line shows the theoretical focal spot sizes given by $\frac{0.514\cdot\lambda}{\mathrm{NA}}$, where λ is the incident wavelength and NA is the numerical aperture with a value of 0.2.

Imaging color objects

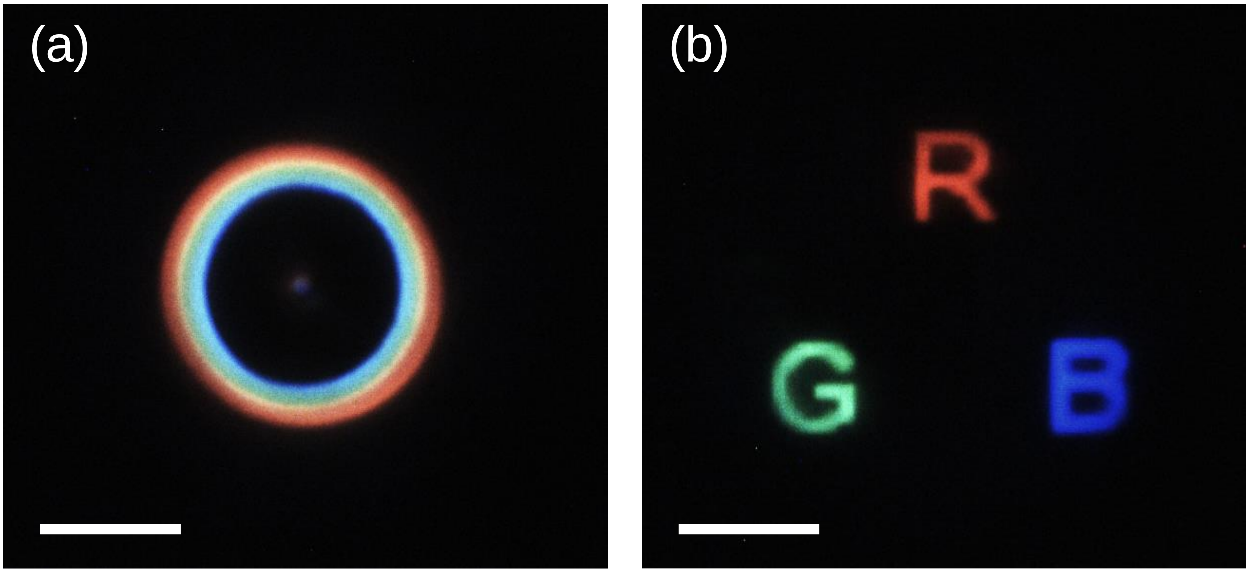

Supplementary Figure 4: Imaging results obtained by an achromatic metalens (NA = 0.05, diameter = 120 μm). (a-b): Images obtained by the achromatic metalens. Scale bar: 3 mm. The objects are pictures on a mobile phone de-magnified 100 times with a microscope objective and then imaged directly by the achromatic metalens on a color camera (Point Gray, GX-FW-28S5C−C).

Library Element Efficiencies

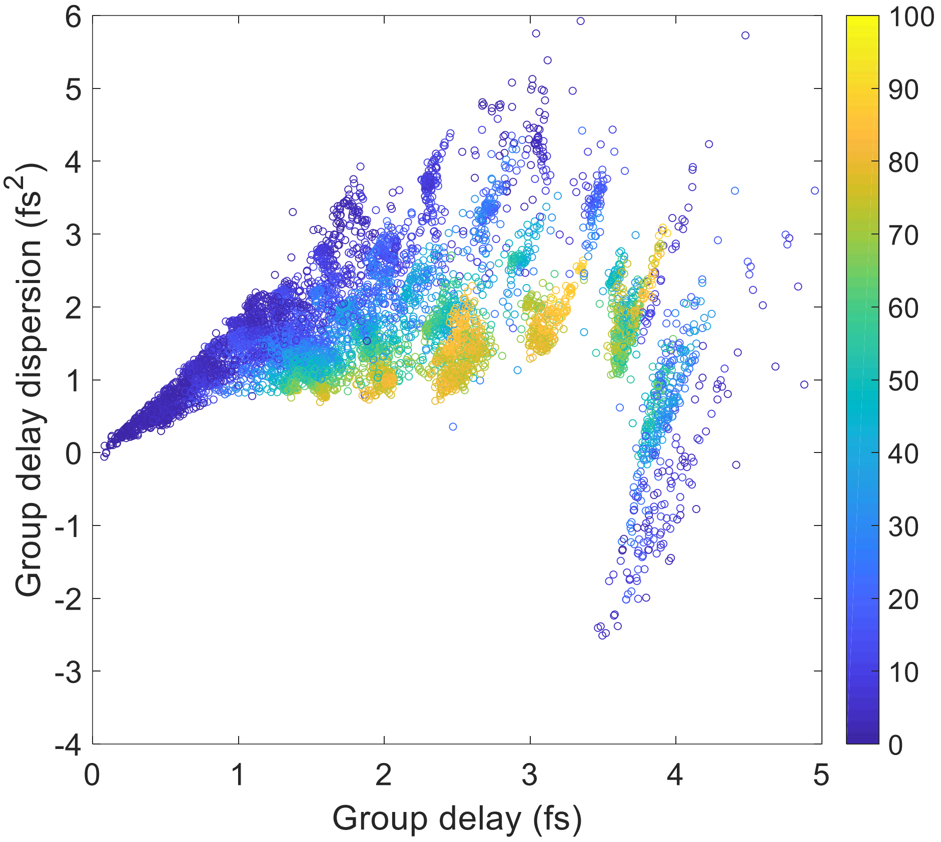

Supplementary Figure 5: Polarization conversion efficiencies of each library element as a function of GD and GDD at the design wavelength, λ_d=560 nm. The color of each dot represents the polarization conversion efficiency (%) of each library element. The polarization conversion efficiency was normalized to the transmitted power through a glass and air interface.

Chromatic Metasurface Beam Deflector

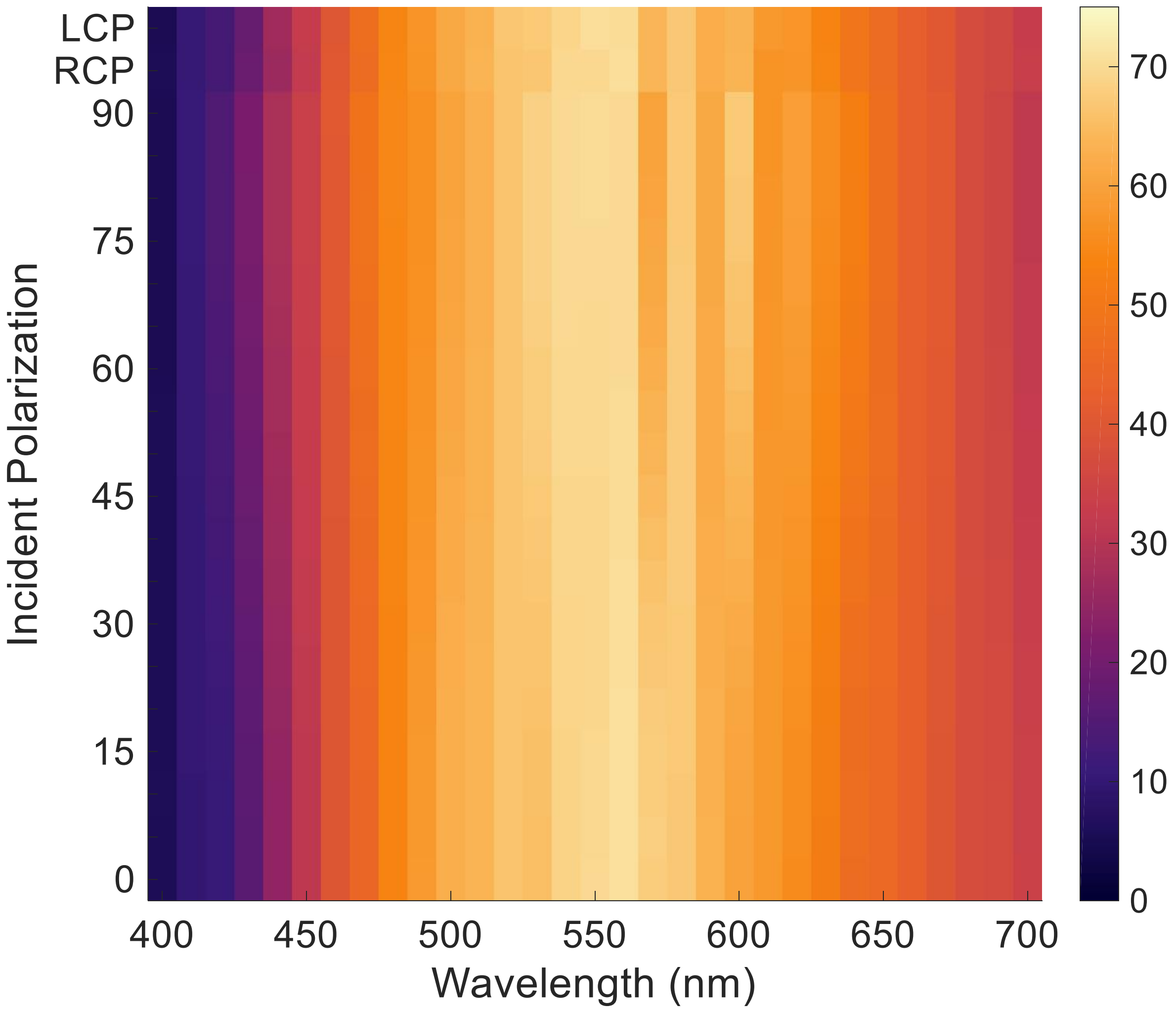

Supplementary Figure 6: Absolute efficiency of a chromatic metasurface beam deflector. The absolute efficiency of the chromatic metasurface beam deflector is shown across the visible spectrum for all linear and both circular incident polarizations. The absolute efficiency is defined by the power diffracted to 15º (1st diffraction order), divided by total incident power.
