# Supplementary material for: A broadband achromatic polarization-insensitive metalens consisting of anisotropic nanostructures
Source: Nat Commun. 2019 Jan 21;10:355. doi: 10.1038/s41467-019-08305-y (PMC6341080; doi:10.1038/s41467-019-08305-y)
Supplement: Supplementary file 3 — Description of Additional Supplementary Files [file 41467_2019_8305_MOESM3_ESM.docx]

**Title:** Supplementary Movie 1

**Description:** This movie was performed by simulating an achromatic and polarization insensitive metalens with a 7.5 μm diameter using an FDTD solver (Lumerical, Inc). The achromatic metalens has a numerical aperture of 0.6. The focusing efficiency of the metalens is given in Supplementary Figure 1. The incident wavelength and polarization are noted in the title of each panel. The last panel in the bottom right corner is a metalens designed without dispersion engineering, showing significant focal length shift.
